# Supplementary material for: Mafosfamide Boosts GMI-HBVac against HBV via Treg Depletion in HBV-Infected Mice
Source: Vaccines (Basel). 2023 May 25;11(6):1026. doi: 10.3390/vaccines11061026 (PMC10303669; doi:10.3390/vaccines11061026)
Supplement: Supplementary file 1 [file vaccines-11-01026-s001.zip › vaccines-2293743-supplementary.pdf]

Supplementary materials

A **Structure of rAAV8-1.3HBV**

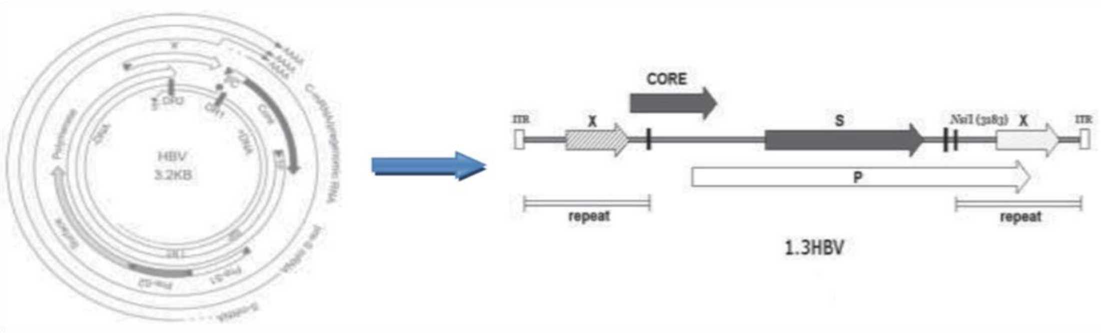

B **MDSCs in chronic hepatitis B mice**

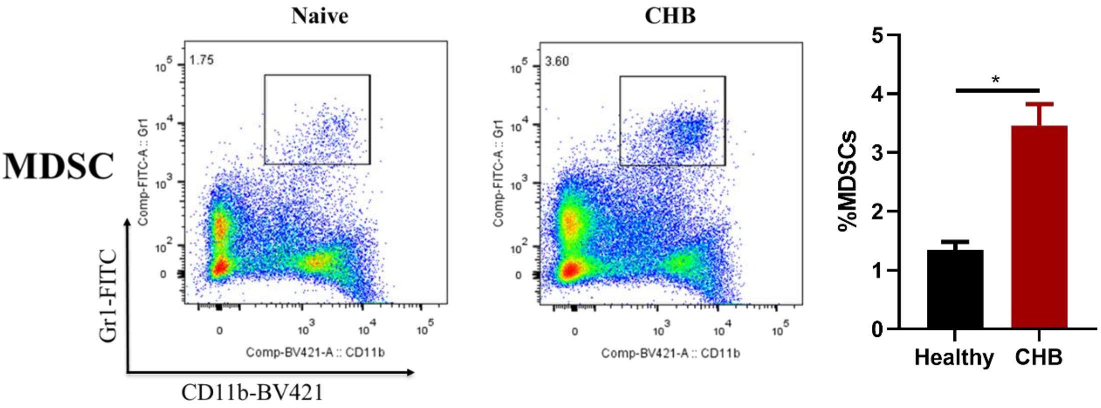

Supplementary 1: structure of rAAV8-1.3HBV(A) and increased MDSCs in rAAV8-1.3HBV infected mice(B).

Screen for optimized concentration of CTX as adjuvant

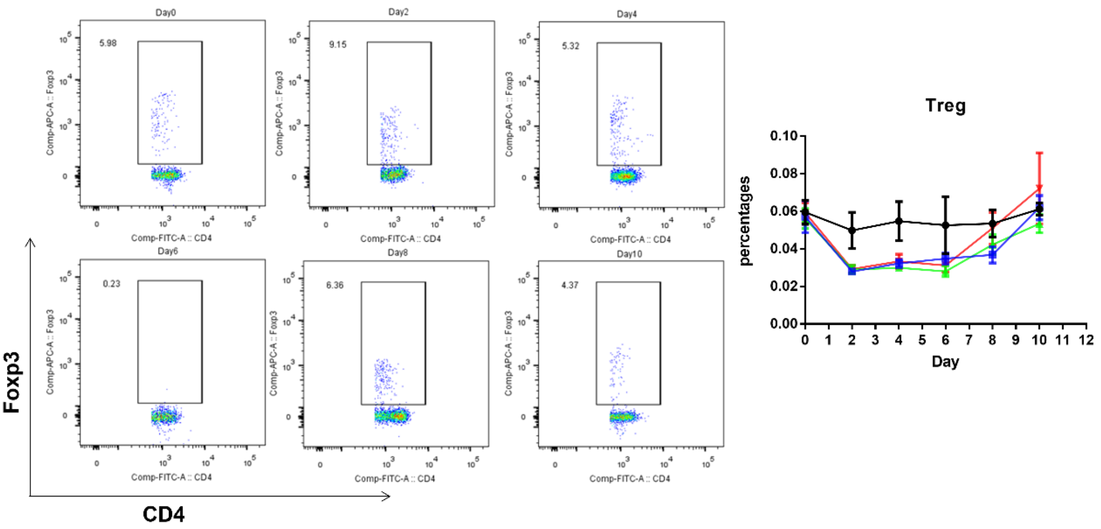

Supplementary 2: Screen for an optimized concentration of CTX as adjuvant.

Footpad(mm)	PBS			GM-CSF+IFN-α+rHBVvac			CTX			CTX+GM-CSF +IFNα+rHBVvac			MAF			MAF+GM-CSF +IFNα+rHBVvac		
24h	0.10	0.04	0.02	0.33	0.32	0.24	0.12	0.16	0.12	0.31	0.34	0.40	0.28	0.38	0.21	0.44	0.48	0.46
48h	0.08	0.11	0.09	0.23	0.19	0.16	0.13	0.14	0.09	0.22	0.23	0.21	0.18	0.26	0.22	0.34	0.38	0.36
72h	0.05	0.08	0.06	0.12	0.08	0.06	0.14	0.13	0.11	0.12	0.14	0.11	0.13	0.20	0.15	0.30	0.27	0.33

Supplementary 3: Footpad of mice after immunization.

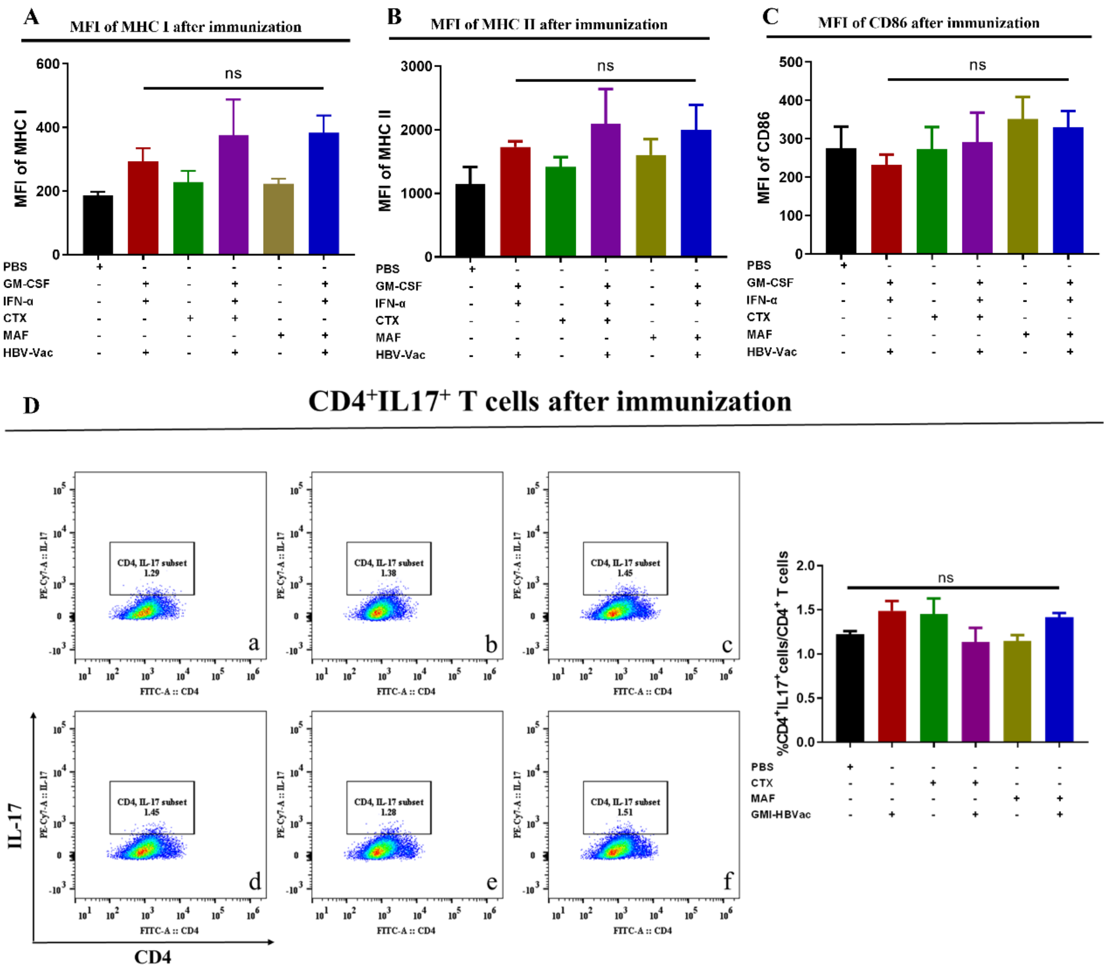

Supplementary 4: costimulatory molecule of DC(A, B, C) and $CD4^{+}IL17^{+}$ cells(D) in peripheral blood after immunization. (a: PBS, b: GMI-HBVac, c: CTX, d: CTX+GMI-HBVac, e: MAF, f: MAF+GMI-HBVac)

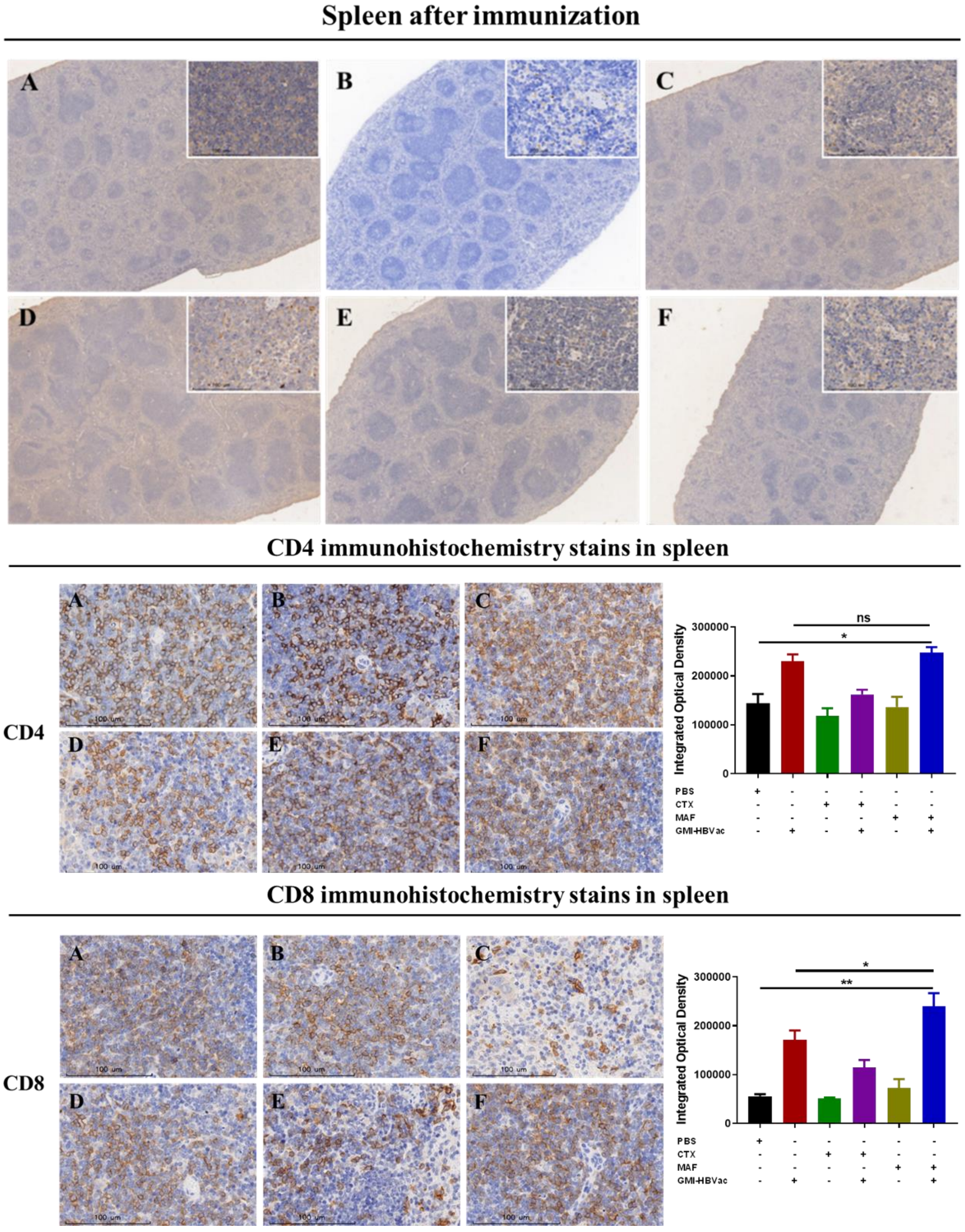

Supplementary 5: Immunohistochemistry stains of CD4 and CD8 in spleen after immunization. (a: PBS, b: GMI-HBVac, c: CTX, d: CTX+GMI-HBVac, e: MAF, f: MAF+GMI-HBVac)
